# Sugar-house Remedy for Consumption

**Published:** 1855-01

**Authors:** 


*Sugar-house Remedy for Consumption.*

Some time since a story passed the rounds of the newspaper press, setting forth that Samuel A. Cartwright, M. D., of New Orleans, had discovered the important fact, that the inhalation of the vapor from the boiling cane-juice in sugar-houses was capable of curing *consumption.* Such a representation purporting to come from an eminent member of the profession, was sufficient to induce a considerable number of sufferers, some of them in the last stages of *Tubercular Phthisis,* to resort to these establishments with a hope of relief. Of course the greater number were entirely disappointed. In a recent number of the Boston Medical and Surgical *Journal*, Dr. Cartwright publishes an article, headed "The sugar-house cure for bronchial, dyspeptic and consumptive complaints;" in which he says: "The author has never recommended the sugar-house remedy as a cure for consumption *in its last and hopeless stages;*" but newspaper misrepresentations and incorrect abridgements, altering the sense more or less of what he did say, have done it for him, and will assuredly bring the sugar-house cure for bronchial. dyspeptic and consumptive complaints into disrepute unless timely corrected. The precise limit of the efficacy of the inhalation of the vapor of boiling cane-juice, in opening the obstructed bronchial tubes and in influencing favorably, their mucous lining, is not yet ascertained; but that it possesses some remarkable virtues in this respect, there is much good evidence to prove."

From the whole tenor of the article before us, we infer that Dr. Cartwright intends to claim for the sugar-house vapor, curative powers only in cases of chronic bronchitis, certain forms of dyspepsia, and the *incipient* stages of *tubercular phthisis;* and not that it has any control over the progress of *tubercular carities* and ulcerations, constituting the advanced stage of true consumption. But he is far from being very explicit in defining what class of cases would be most certainly benefitted by a resort to the proposed remedy. The subject is worthy of a careful investigation by those members of the profession who have abundant opportunities.

